# Supplementary material for: PABPN1 gene therapy for oculopharyngeal muscular dystrophy
Source: Nat Commun. 2017 Mar 31;8:14848. doi: 10.1038/ncomms14848 (PMC5380963; doi:10.1038/ncomms14848)
Supplement: Supplementary Information — Supplementary figures and supplementary note. [file ncomms14848-s1.pdf]

Supplementary Information

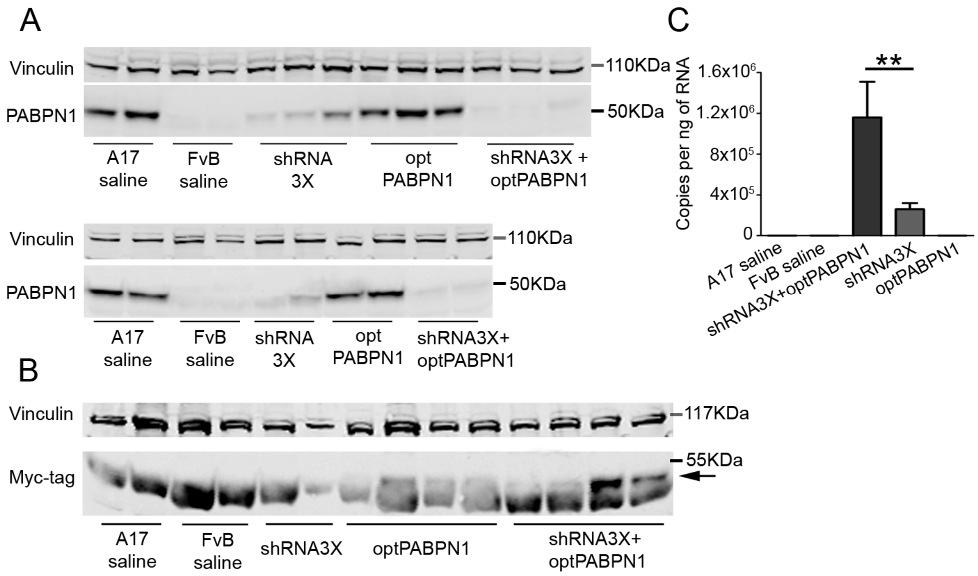

Supplementary figure 1: ***Tricistronic shRNA delivered in vivo by AAV vectors efficiently down-regulates endogenous PABPN1 without affecting optPABPN1 expression in vivo.***

(a) Western blot for PABPN1 expression on the remaining TA muscles shows that the treatments with AAV-shRNA3X alone or in combination with AAV-optPABPN1 significantly inhibited the endogenous PABPN1 and that the effect was consistent in all treated muscles: n = 4 (saline treated A17 or FvB muscles) or n=5 (all the other groups). **(b)** Representative western blot showing the detection of MYC-tag in the samples not included in Figure 2E: n = 2 (saline treated A17 or FvB muscles and shRNA3X treated muscles) or n=4 (the other two groups). The arrow shows the band detected at the correct molecular weight. **(c)** Analysis by qRT-PCR shows a significant expression of shRNAs in muscles treated either with the tricistronic cassette or the tricistronic cassette and optPABPN1. Data are represented as mean ± SEM: n = 8. One-way Anova test with Bonferroni post-doc test **p<0.01.

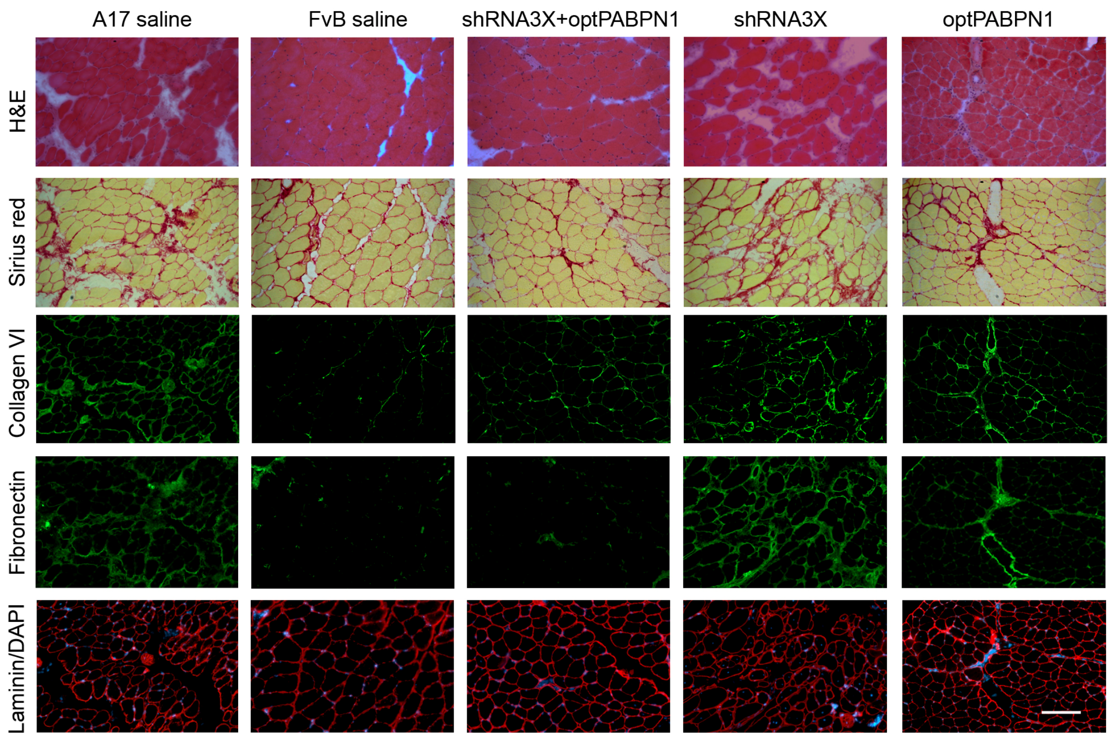

Supplementary figure 2: ***In vivo administration of AAVs expressing shRNA3X and optPABPN1 reduces muscle fibrosis.*** Representative images in serial sections of histological stainings for Hematoxylin & Eosin and Sirius red and immunostaining for Collagen VI, Fibronectin and Laminin/DAPI. Several centrally nucleated fibres are shown by both H&E and Laminin/DAPI stainings in shRNA3X treated muscles demonstrating that muscle degeneration/regeneration process is ongoing in these muscles. Muscles treated with shRNA3X and optPABPN1 show a remarkable reduction in Collagen I, III (by Sirius red), Collagen VI and Fibronectin compared to saline injected A17 muscles. Bar, 200 μm

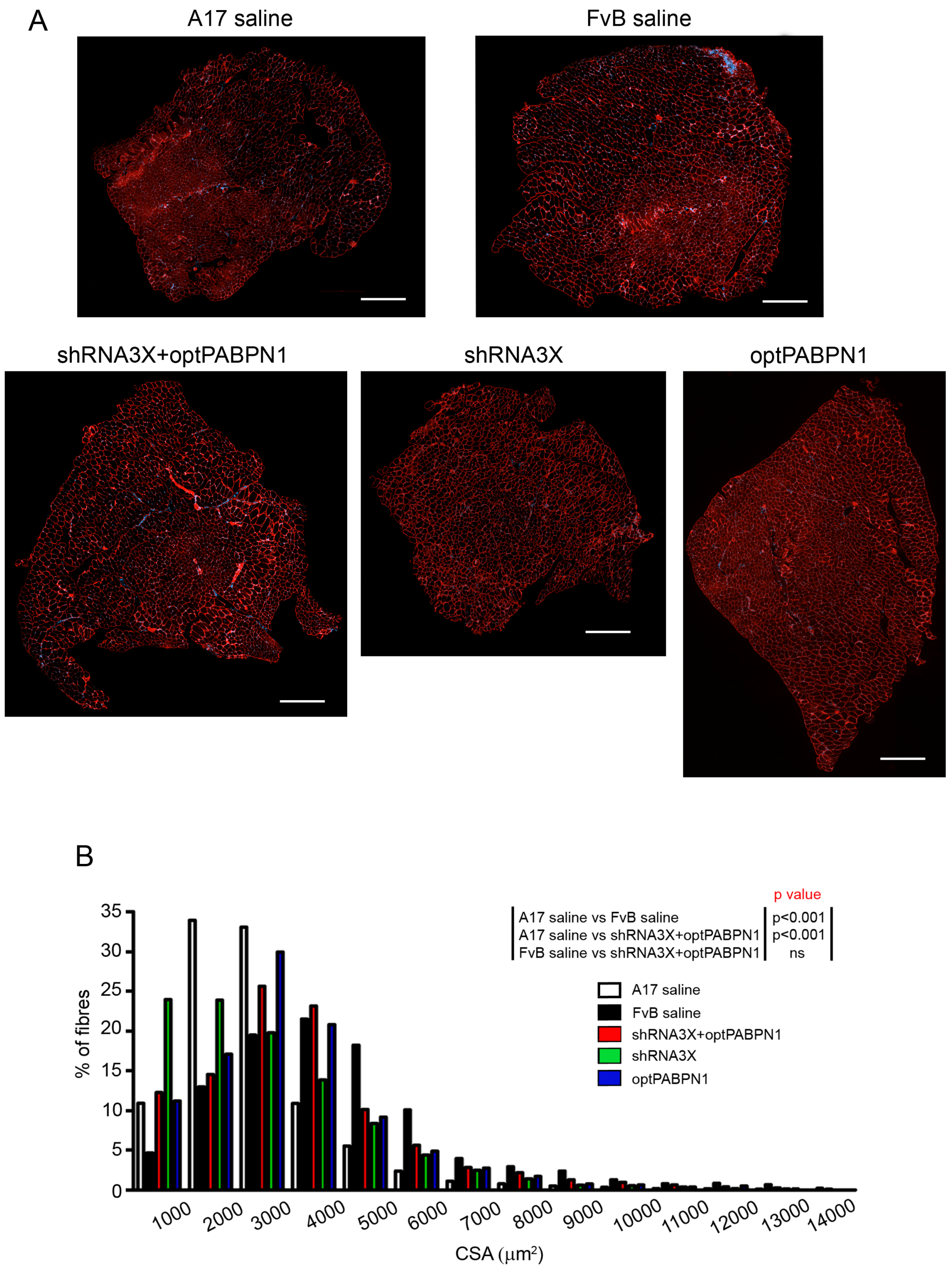

Supplementary figure 3*: Distribution of myofibre cross sectional area (CSA) in treated muscles.* **(a)** Laminin/Dapi stainings of representative full cross sections of TA muscles showing that although the presence of variability between fibres in the inner and in the outer regions of

the muscle, the treatment with the two vectors induced a small but detectable increase in cross section area of muscles. Bar, 500 µm. **(b)** Comparison of different groups by Chi-squared analysis indicates changes in myofibre distribution for muscles treated with AAV vectors compared with saline injected A17 muscles (A17 saline vs FvB saline, χ^2=79.1; A17 saline vs shRNA3X, χ^2=30.3; A17 saline vs optPABPN1, χ^2=27.2; A17 saline vs shRNA3X+optPABPN1, χ^2=44; FvB saline vs shRNA3X+optPABPN1, χ^2=22. Freedom degrees=13. All groups, n = 6-8.

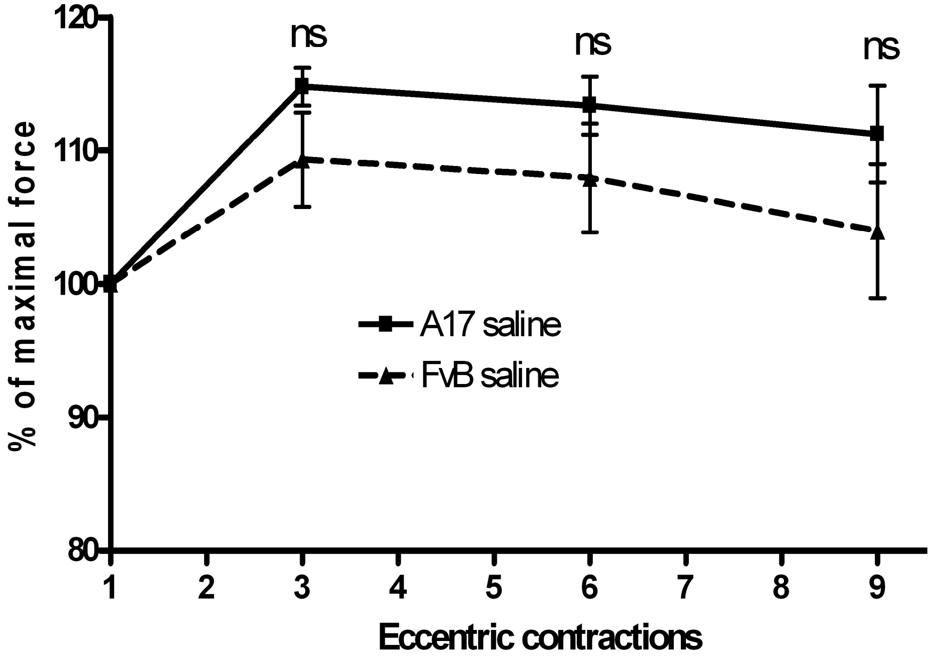

Supplementary figure 4*: Resistance to eccentric contractions is not affected in A17 compared to wild type muscles.* Resistance generated by TA muscles of A17 and FvB muscles to 9 eccentric contractions was measured by *in situ* muscle physiology: no decrease was observed in saline treated A17 muscles compared with wild type FvB muscles. n=6 (both groups). Unpaired t-test, ns: not significant.

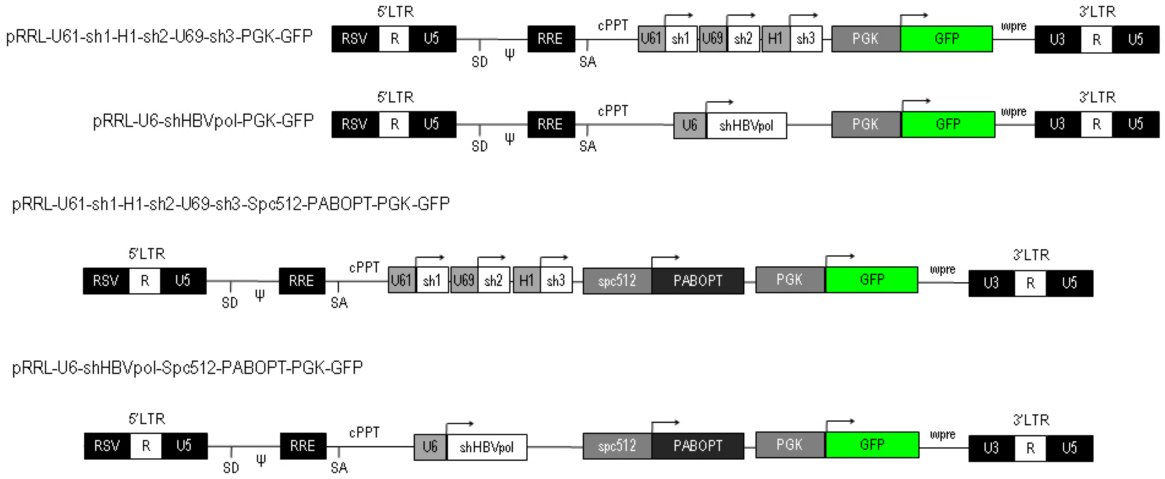

Supplementary figure 5*: LV contructs used for the transduction of HEK293T cells and human OPMD myoblasts.* LV plasmid vectors expressing tricistronic shRNA together with a PGK-GFP reporter cassette with or without SPc512-PABOPT.

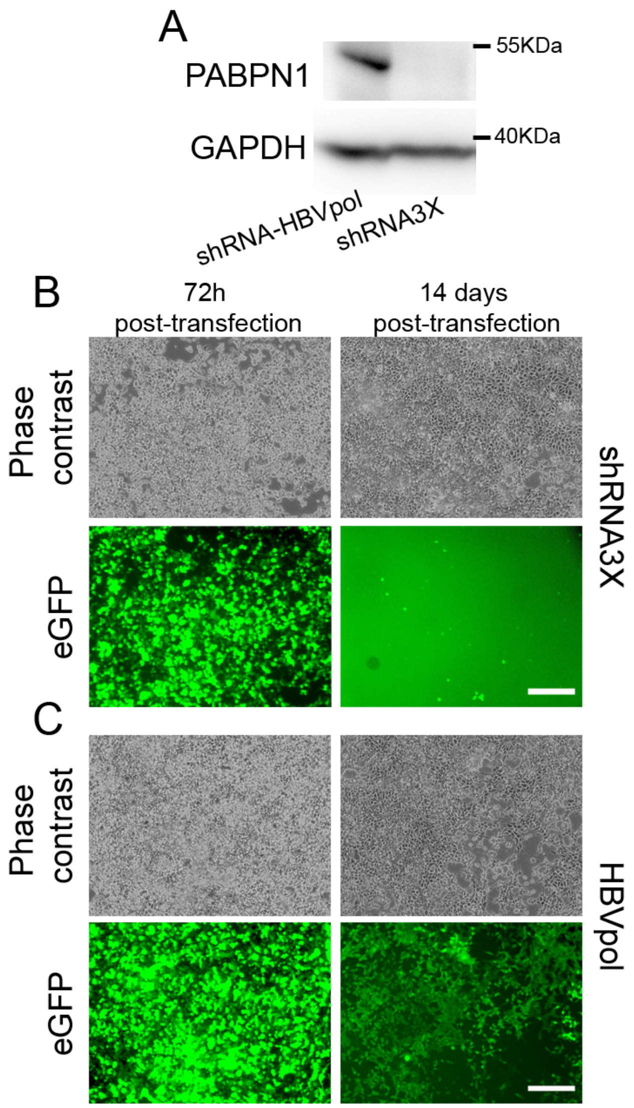

Supplementary figure 6*: PABPN1 inhibition in human HEK293T cells induces cell death that can be prevented by optPABPN1 co-expression.* **(a)** HEK293T cells are cotransfected with expPABPN1 expression vector together with the shRNA3X construct and expPABPN1 knockdown was confirmed at protein level by western-blot. **(b-c)** Lentiviral constructs contained a GFP reporter cassette, so GFP expression was followed at 72 h and 2 weeks after transfection in HEK293T cells by fluorescent microscopy. No GFP positive HEK293T cells survived 2 weeks after transfection with shRNA3X LV construct. Bar, 100 µm.

Figure 1c

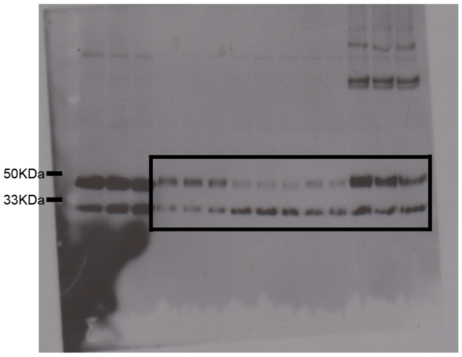

Figure 1d

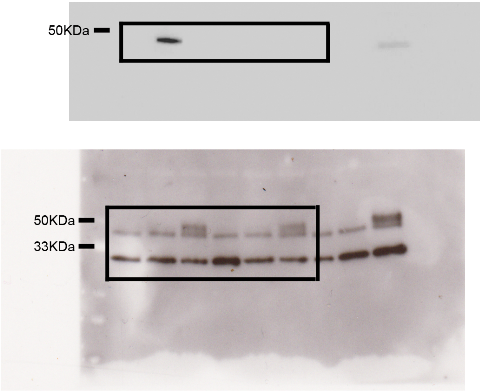

Figure 2b

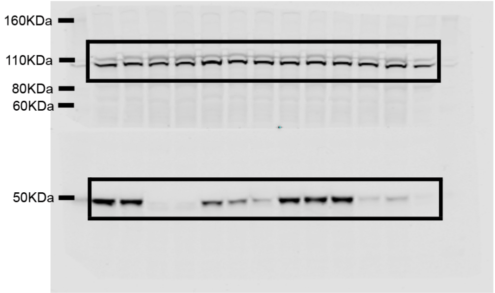

Figure 2e

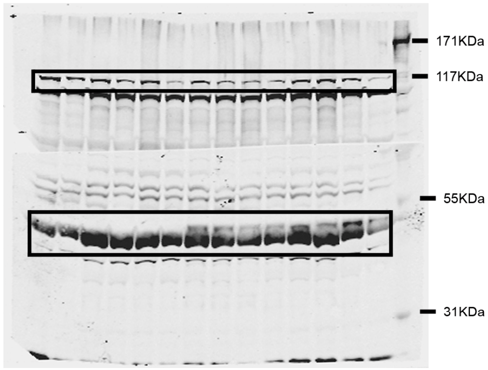

Supplementary Figure 1a

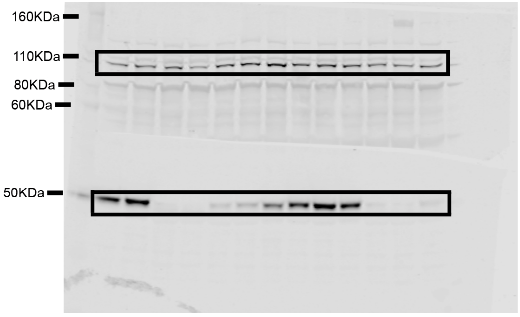

Supplementary Figure 1b

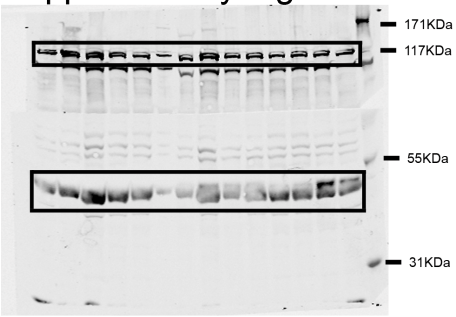

Supplementary Figure 1a

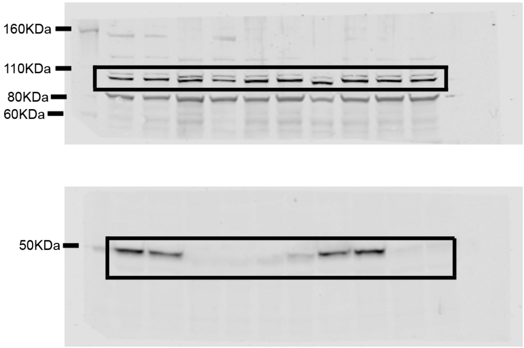

Supplementary Figure 6a

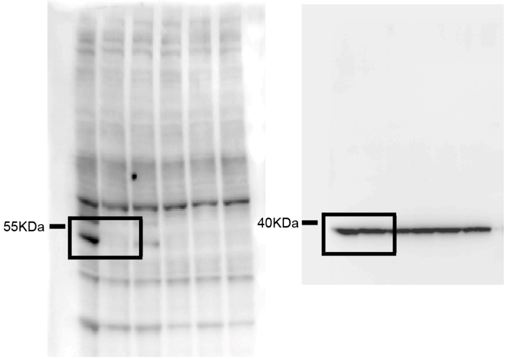

Supplementary figure 7*: uncropped western blot included on this study*

Supplementary Note 1

shRNA3X construct sequence

ggcaggaagagggcctatttcccatgattccttcatatttgcatatacgatacaaggctgttagagagataattagaattaatttgactgtaaaca
caaagatattagtacaaaatacgtgacgtagaaagtaataatttcttgggtagtttgcagttttaaaattatgttttaaaatggactatcatatgctta
ccgtaacttgaaagtatttcgatttcttggctttatatatcttgtggaaaggacgaaacaccgaggagaagatggaggctgatcaagagaatca
gcctccatcttctcctctttttttacatcaggttgtttttctgtttttacatcaggttgtttttctgtttggtttttttttacaccacgtttatacgccggtgca
cggtttaccactgaaaacacctttcatctacaggtgatatcttttaacacaaataaaatgtagtagtccatgcgcacgtttaaacaagggcggtg
cggctcaggctctgccccgcctccggggctatttgcatacgaccatttccagtaattcccagcagccaccgtagctatatttggtagaacaac
gagcactttctcaactccagtcaataactacgttagttgcattacacattgggctaatataaatagaggttaaatctctaggtcatttaagagaag
tcggcctatgtgtacagacatttgttccaggggctttaaatagctggtggtggaactcaatattcggaagaagctgagaagctaacaagagat
tagcttctcagcttcttcctttttttccattttccctcccagaaacggaatcttgctctgttgcccaggctggaatgcaatggcgcgatcttagcttgt
tgcaacctccgcctcccgggttgaagcgattctcctgcctcagcctcccgagtagctgggattataaacatgcgccagtatacgatacctatc
aattcgaacgctgacgtcatcaacccgctccaaggaatcgcgggcccagtgtcactaggcgggaacacccagcgcgcgtgcgccctggc
aggaagatggctgtgagggacaggggagtggcgccctgcaatatttgcatgtcgctatgtgttctgggaaatcaccataaacgtgaaatgtc
tttggatttgggaatcttataagttctgtatgagaccacagatccccgaggtagagaagcagatgaatatgagttcaagagactcatattcatct
gcttctctacctctttttaaacaaaacgaaaccgggccgggcgcggtggttcacgcctataatccctgcactttgggaggccgaggcgggc
ggatcacaaggtcaggaggtcgagaccatccaggctaacacggtgaaaccccccccatctctactaaaaaaaaaaa

U61 Promoter

U69 Promoter

H1 Promoter

shRNA1 – shRNA2 – shRNA3

Codon optimized human PABPN1 sequence

cgagctccaccgcggtggcggccgtccgccctcggcaccatcctcacgacacccaaatatggcgacgggtgaggaatggtggggagtt
atttttagagcggtgaggaaggtgggcaggcagcaggtgttggcgctctaaaaataactcccgggagttatttttagagcggaggaatggtg
gacacccaaatatggcgacggttcctcacccgtcgccatatttgggtgtccgccctcggccggggccgcattcctgggggccgggcggtg
ctcccgcccgcctcgataaaaggctccggggccggcggcggcccacgagctacccggaggagcgggaggcgccaagctctagaacta
gtggatcccccgggctgcaggaattctgcagtcgacggtaccgatatcgccatggccgctgccgccgctgctgctgccgcagccggcgct
gccggcggaagaggcagcggccctggcagacggcggcatctggtccctggcgccggaggggaggccggcgaaggcgcccctggcg
gagccggcgactacggcaacggcctggaaagcgaggaactggaacccgaggaactgctgctggaacctgagcccgagccagagccc

gaggaagagccccctaggccaagagcccccccctggcgccccaggaccaggaccaggctctggggcaccaggctctcaggaagagga
agaagagcccggcctcgtcgagggagacccaggcgatggcgctatcgaagatcccgagctggaagccatcaaggccagagtgcggga
gatggaagaggaggccgaaaaattgaaagagctgcagaacgaagtcgaaaaacaaatgaacatgtcccccccctcctggaaatgctggcc
ctgtgatcatgagcatcgaggaaaagatggaagccgacgcccggtctatctacgtgggcaacgtggactacggcgccaccgccgaagaa
ctggaagcccactttcacggctgtggcagcgtgaaccgggtgaccatcctgtgcgacaagttcagcggccaccccaagggcttcgcctac
atcgagttcagcgacaaagaaagcgtgcggacctctctggctctcgacgagtctctgttcaggggaaggcagatcaaggtcatccccaag
cggaccaacaggcccggcatcagcaccaccgacagaggcttccctagggctaggtacagagcccggaccaccaactacaacagcagc
agaagccggttctacagcggcttcaattctcggcctagaggcagagtgtaccggggcagggccagggccacctcctggtacagcccta
cgaacagaagctgatcagcgaggaagatctgtgatgagatatc

spc5-12 promoter

optPABPN1 sequence

Start codon

Myc-tag sequence

expanded human PABPN1 sequence

cgagctccaccgcggtggcggccgtccgccctcggcaccatcctcacgacacccaaatatggcgacgggtgaggaatggtggggagtt
atttttagagcggtgaggaaggtgggcaggcagcaggtgttggcgctctaaaaataactcccgggagttatttttagagcggaggaatggtg
gacacccaaatatggcgacggttcctcacccgtcgccatatttgggtgtccgccctcggccggggccgcattcctgggggccgggcggtg
ctcccgcccgcctcgataaaaggctccggggccggcggcggcccacgagctacccggaggagcgggaggcgccaagctctagaacta
gtggatcccccgggctgcaggaattctgcagtcgacggtaccatggcggcggcggcggcggcagcagcagcgggggctgcggg
cggtcggggctccgggccggggcggcggcgccatcttgtgcccggggccggtggggaggccggggagggggccccgggggcgc
aggggactacgggaacggcctggagtctgaggaactggagcctgaggagctgctggagcccgagccggagcccgagcccgaag
aggagccgccccggccccgcgcccccccgggagctccgggccctgggcctggttcgggagcccccggcagccaagaggaggagga
ggagccgggactggtcgagggtgacccgggggacggcgccattgaggacccggagctggaagctatcaaagctcgagtcagggagat
ggaggaagaagctgagaagctaaaggagctacagaacgaggtagagaagcagatgaatatgagtccacctccaggcaatgctggcccg
gtgatcatgtccattgaggagaagatggaggctgatgcccgttccatctatgttggcaatgtggactatggtgcaacagcagaagagctgga
agctcactttcatggctgtggttcagtcaaccgtgttaccatactgtgtgacaaatttagtggccatcccaaagggtttgcgtatatagagttctc
agacaaagagtcagtgaggacttccttggccttagatgagtccctatttagaggaaggcaaatcaaggtgatcccaaaacgaaccaacaga
ccaggcatcagcacaacagaccggggttttccacgagcccgctaccgcgcccggaccaccaactacaacagctcccgctctcgattctac
agtggttttaacagcaggccccggggtcgcgtctacaggggccgggctagagcgacatcatggtattccccttacgactacaaggacgac
gatgacaag

spc5-12 promoter

expPABPN1 sequence

Start codon

FLAG sequence

Primers

PABPN1-FWD 5’-TGACCCGGGGGACGGCGC-3’

PABPN1-REV 5’-ACTCGAGCTTTGATAGCTTCCAGC-3’

optPABPN1-FWD 5’-GGCACCAGGCTCTCAGGAA-3’

optPABPN1-REV 5’-GGGATCTTCGATAGCGCCA-3’

MYH3-FWD 5’-TCACCAAGTTCAGGAAAGCC-3’

MYH3-REV 5’-CTCGCTTTCATGGACCACCA-3’

RPLP0-FWD 5’-GAGGACCTCACTGAGATTCGG-3’

PRLP0-REV 5’-TTCTGAGCTGGCACAGTGAC-3’

OPMD_Hairpin A 5’-ATCAGCCTCCATCTTCTCCTC-3’

OPMD_Hairpin F 5’-TTAGCTTCTCAGCTTCTTCC-3’

OPMD_Hairpin G 5’-TCATATTCATCTGCTTCTCTA-3’
